# Research on Dynamic Strength and Inertia Effect of Concrete Materials Based on Large-Diameter Split Hopkinson Pressure Bar Test

**DOI:** 10.3390/ma15092995

**Published:** 2022-04-20

**Authors:** Bi Sun, Rui Chen, Yang Ping, ZhenDe Zhu, Nan Wu, Zhenyue Shi

**Affiliations:** 1 Harbin Institute of Technology (Shenzhen), Shenzhen 518055, China; sunbi58@126.com (B.S.); cechenrui@hit.edu.cn (R.C.); 2 Shenzhen Water Planning and Design Institute Co., Ltd., Shenzhen 518001, China; 3 PowerChina Eco-Environment Group Co., Ltd., Shenzhen 518102, China; pingy@swpdi.com; 4 Key Laboratory of Ministry of Education of Geomechanics and Embankment Engineering, Hohai University, Nanjing 210098, China; zhendezhunj@163.com; 5 Guangzhou University-Tamkang University Joint Research Centre for Engineering Structure Disaster Prevention and Control, Guangzhou University, Guangzhou 510006, China; wunan@gzhu.edu.cn; 6 College of Safety and Environmental Engineering (College of Safety and Emergency Management), Shandong University of Science and Technology, Qingdao 266590, China

**Keywords:** large-diameter SHPB, high strain rate, concrete material, strain rate effect, inertia effect, dynamic strength

## Abstract

The Split Hopkinson Pressure Bar (SHPB) test device is an important tool to study the dynamic characteristics of concrete materials. Inertial effect is one of the main factors that cause inaccurate results in SHPB tests of concrete materials. To solve this problem, Large-diameter SHPB tests on concrete and mortar were performed. A dynamic increase factor (DIF) model considering strain rate effect and inertia effect was established. This model provides a scientific reference for studying the dynamic mechanical properties of concrete materials. The experimental results indicate that the strain rate effect of concrete is more sensitive than that of mortar, but the inertia effect of mortar is more sensitive than that of concrete. Under the same strain rate, the energy utilization rate, average fragment size, and impact potentiality of mortar are higher than concrete.

## 1. Introduction

Concrete materials, the largest and most widely used engineering building materials, are affected by static loads and high strain rate dynamic loads such as earthquakes, impacts, and explosions. As a heterogeneous, anisotropic, and strain rate-sensitive multiphase composite, concrete will show more complex dynamic mechanical characteristics under dynamic load than under static load, which has been one of the hot topics in recent years. Split Hopkinson Pressure Bar (SHPB) experimental technology is the most commonly used test method for the dynamic performance of concrete [1,2,3,4,5,6,7]. For heterogeneous materials, such as concrete, a specimen of considerable size is required to ensure a certain degree of homogeneity because concrete has a large aggregate size and has many microscopic defects. Ensuring a certain homogeneity in the SHPB test requires a fairly large specimen size. The pressure bar diameter of the SHPB device should also be increased correspondingly [8,9,10]. In the large-diameter SHPB test, the dynamic strength of the specimen is affected by the strain rate effect [11,12,13] and the inertia effect [14].

The dynamic increase factor (DIF), defined as the ratio of dynamic-to-static strength, is conventionally considered a material property [15,16]. A bilinear DIF model proposed by the Comité Euro-International du Béton (CEB) and the Fédération International de la Précontrainte (FIP) (CEB-FIP) [17] standard is the most representative achievement. Tedesco et al. [18] conducted SHPB tests on concrete specimens with a diameter and length of 51 mm, and presented a regression equation to describe the relationship between the DIF and log10 of strain rate. Based on the SHPB test of concrete and mortar, Grote et al. [19] suggested a nonlinear DIF model at a high strain rate with a 250~1700 s^−1^ range. Ngo et al. [20] established a new relationship model between DIF and strain rate. The model considers the effects of various factors on dynamic strength and is suitable for concrete with a strength range of 32–160 MPa. Katayama et al. [21] adopted a quadratic equation of logarithmic strain rate to express the DIF model by introducing it into Drucker–Prager’s equation. Hartmann et al. [22] used a power function to describe the relationship between DIF and strain rate.

With the progress of technology and the deepening of research, some scholars found that under high strain rate [23,24], the inertial effect is not eliminated, but dominant [25,26]. Under the axial dynamic load, part of the work of the external load is to provide kinetic energy to the particles so that the particles obtain axial acceleration. Due to the Poisson effect, particles will interact with adjacent particles, which will obtain radial acceleration. The load that provides acceleration to the particles is the inertial effect, which is part of the macro-bearing capacity [27]. Gorham [28] provided a relatively perfect inertia effect correction model based on the energy conservation law. Forrestal [14] also proved the existence of the inertia effect in theory. Guo et al. [29] considered that the radial inertia effect is significant only when the strain rate exceeds 110 s^−1^. Flores-Johnson et al. [30] believed that the lateral confinement effect of the SHPB specimen is the main reason for the structural effect of all concrete-like materials. Li et al. [31] considered that the lateral inertial confinement of a cylindrical specimen was higher than a cubic specimen at the same strain rate. Zhou et al. [32] expressed that the increase of material strength is due to the inertia effect rather than strain rate effect. A quadratic equation was used to describe the relationship between DIF and the log of the strain rate and quantitatively confirmed by Li et al. [33,34]. Hao et al. [35,36] proposed that the reason for the large discreteness of the experimental results is that the inertial effect is unaccounted for. The quadratic equation was used to express the relationship between DIF and log of the strain rate. Xu et al. [37] presented semi-empirical equations for the concrete material DIF considering the internal configuration effect, by adopting a hyperbolic tangent function. Al-Salloum [38] used a power function to express the DIF model. Lu et al. [27] established a nonlinear dynamic uniaxial strength criterion, called the S criterion, based on understanding the physical mechanisms. Lee et al. [39,40] described pure rate DIF with strain rate and inertial effect with strain acceleration. The sum of the two obtains apparent DIF.

Inertia effect is an important factor causing the inaccuracy of SHPB test results of concrete materials. In order to explore the influence of the inertia effect on the strength of concrete materials under the dynamic load, SHPB tests of concrete and mortar were carried out in this paper. A DIF model considering strain rate effect and inertia effect was established. The dynamic mechanical response characteristics of mortar and concrete were compared and analyzed, which provides a theoretical basis and scientific support for seismic design and safety evaluation of concrete engineering.

## 2. Research on Strain Rate and Inertia Effect

### 2.1. Strain Rate Effect Research

Some typical empirical formulas for DIF have been developed based on the SHPB test of mortar and concrete specimens. The formulae were either based on power-law variation or followed the logarithmic trends [38]. One of the most commonly used empirical formulas for DIF was given by the CEB [17]. The DIF of the strain rate-dependent behavior of mortar and concrete can be obtained by the following piecewise function:

(1) $$\mathrm{DIF}=\frac{f_{cd}}{f_{cs}}=\left\{ \begin{array}{l} {\left(\dot{\varepsilon }/{\dot{\varepsilon }}_{s}\right)}^{1.026\alpha_{s}}\;\;\;\left|\dot{\varepsilon }\right|\leq 30\;s^{-1}\\ \gamma_{s}{\left(\dot{\varepsilon }/{\dot{\varepsilon }}_{s}\right)}^{1/3}\;\;\;\;\left|\dot{\varepsilon }\right|>30\;s^{-1} \end{array}\right.$$

where $\dot{\varepsilon }$ is the strain rate, $f_{cs}$ and $f_{cd}$ are the unconfined compressive strength in quasi-static and dynamic loading, respectively, and $\gamma_{s}=10^{\left(6.156\alpha_{s}-2.0\right)}$, $\alpha_{s}=1/(5+9f_{cs}/f_{c0})$, $f_{c0}=10\;\mathrm{MPa}$, ${\dot{\varepsilon }}_{s}=30\times 10^{-6}\;/s$. Equation (1) shows a nonlinear relationship between the dynamic strength of mortar and concrete and the high strain rate.

Tedesco and Ross [18] conducted a series of SHPB tests where the DIF rapidly increases with the strain rate. A logarithmic function can describe the relationship between DIF and strain rate:

(2) $$\mathrm{DIF}=\left\{ \begin{array}{l} 0.00965\log\dot{\varepsilon }+1.058\geq 1.0\;\;\;\dot{\varepsilon }\leq 63.1\;s^{-1}\\ 0.758\log\dot{\varepsilon }-0.289\leq 2.5\;\;\;\;\;\;\dot{\varepsilon }>63.1\;s^{-1} \end{array}\right.$$

Through explosion resistance tests of the ultra-high-strength concrete panel, Ngo et al. [20] also believed that there is a logarithmic relationship between DIF and strain rate at high strain rates with the formula:

(3) $$\mathrm{DIF}=\frac{f_{cd}}{f_{cs}}=\left\{ \begin{array}{l} {\left(\frac{\dot{\varepsilon }}{{\dot{\varepsilon }}_{s}}\right)}^{1.026\alpha }\;\;\;\;\;\;\;\;\dot{\varepsilon }\leq {\dot{\varepsilon }}_{1}\\ A_{1}\ln(\dot{\varepsilon })-A_{2}\;\;\;\dot{\varepsilon }>{\dot{\varepsilon }}_{1} \end{array}\right.$$

where ${\dot{\varepsilon }}_{s}=3\times 10^{-5}s^{-1}$, $\alpha =1/(20+f_{cs}/2)$, ${\dot{\varepsilon }}_{1}=0.0022f_{cs}^{2}-0.1989f_{cs}+46.137$, $A_{1}=-0.0044f_{cs}+0.9866$, $A_{2}=-0.0128f_{cs}+2.1396$.

Guo et al. [29] viewed that the CEB-FIP 2010 equation [41] is not suitable for high strength concrete through the SHPB test of concrete with different strength, and proposed the following formula:

(4) $$\mathrm{DIF}=\left\{ \begin{array}{l} {\left(\dot{\varepsilon }/{\dot{\varepsilon }}_{s}\right)}^{0.014}\;\;\;\dot{\varepsilon }\leq {\dot{\varepsilon }}_{TR}\\ A\log_{10}(\dot{\varepsilon }/{\dot{\varepsilon }}_{s})+B\;\;\;\;\dot{\varepsilon }>{\dot{\varepsilon }}_{TR} \end{array}\right.$$

where ${\dot{\varepsilon }}_{TR}$ is the transition strain rate, and A and B are constants.

### 2.2. Inertial Effect Research

Because the linear function cannot accurately describe the relationship between the DIF and the logarithmic strain rate under high a strain rate, some scholars used quadratic or cubic equations to describe it. Grote et al. [19] tested the cement mortar specimens on SHPB with strain rates ranging from 250 to 1700 s^−1^ and gave the following equations:

(5) $$\mathrm{DIF}=\left\{ \begin{array}{l} 0.0235\log\dot{\varepsilon }+1.07\geq 1.0\;\;\;\dot{\varepsilon }\leq 266.0\;s^{-1}\\ 0.882(\log\dot{\varepsilon }{)}^{3}-4.4{\left(\log\dot{\varepsilon }\right)}^{2}+7.22(\log\dot{\varepsilon })-2.64\;\;\;\dot{\varepsilon }>266.0\;s^{-1} \end{array}\right.$$

Li et al. [34] conducted experimental and numerical studies on mortar samples. Their research results confirmed quantitatively that the apparent dynamic strength enhancement of concrete-like materials in a SHPB test is caused by the lateral inertia confinement instead of the strain rate sensitivity of the tested material. The DIF model was proposed as:

(6) $$\mathrm{DIF}=\left\{ \begin{array}{l} 0.03438(\log\dot{\varepsilon }+3)+1\;\;\;\;\dot{\varepsilon }\leq 100\;s^{-1}\\ 1.729(\log\dot{\varepsilon }{)}^{2}-7.1372\log\dot{\varepsilon }+8.5303\;\;\dot{\varepsilon }>100\;s^{-1} \end{array}\right.$$

Katayama et al. [21] believed that if the mass is retained, the inertia conservation and the spatial continuity of inertia can be maintained and presented another DIF model as:

(7) $$\mathrm{DIF}=0.2583(\log\dot{\varepsilon }{)}^{2}-0.05076\log\dot{\varepsilon }+1.021$$

Hao et al. [35,36] regarded that the friction at the sample bar interface is an important factor affecting the lateral inertia effect of the specimen under high-speed impact. They proposed an empirical formula to remove the influence of end friction confinement on dynamic strength increment of concrete material as:

(8) $${\mathrm{DIF}}_{Mortar}=\left\{ \begin{array}{l} 0.0419\log\dot{\varepsilon }+1.2165\;\;\;\;\;\;\;\;1\;s^{-1}<\dot{\varepsilon }\leq 10\;s^{-1}\\ 0.8988{\left(\log\dot{\varepsilon }\right)}^{2}-2.8255(\log\dot{\varepsilon })+3.4907\;\;\;30\;s^{-1}<\dot{\varepsilon }\leq 1000\;s^{-1} \end{array}\right.$$

(9) $${\mathrm{DIF}}_{Aggregate}=\left\{ \begin{array}{l} 0.0191\log\dot{\varepsilon }+1.2222\;\;\;\;\;\;\;\;1\;s^{-1}<\dot{\varepsilon }\leq 220\;s^{-1}\\ 1.6607{\left(\log\dot{\varepsilon }\right)}^{2}-6.9122(\log\dot{\varepsilon })+8.346\;\;\;220\;s^{-1}<\dot{\varepsilon }\leq 1000\;s^{-1} \end{array}\right.$$

where ${\mathrm{DIF}}_{Aggregate}$ is the DIF of concretes with aggregates.

Under a high strain rate, the inertia effect cannot be eliminated [23], and it dominates [26,29]. However, there is no further study on the influence of the inertia effect in Equations (5)–(9). Quadratic or cubic equations were used to fit the experimental data to obtain a higher fitting degree, leading to a non-conservative prediction [39]. Lee et al. [39,40] proposed a new concrete DIF that excludes inertia effects by considering the strain acceleration and geometry of the specimens based on SHPB test results, described by the formula:

(10) $$\left\{ \begin{array}{l} {\mathrm{DIF}}_{apparent}{=\mathrm{DIF}}_{rate}+\Delta f_{inertia}\\ {\mathrm{DIF}}_{rate}={\left(\frac{\dot{\varepsilon }}{{\dot{\varepsilon }}_{s}}\right)}^{k_{1}}\\ \Delta f_{inertia}=k_{2}\frac{\rho_{s}d_{s}^{2}}{f_{cs}}\ddot{\varepsilon }+k_{3}\frac{\rho_{s}l_{s}^{2}}{f_{cs}}\ddot{\varepsilon } \end{array}\right.$$

where ${\mathrm{DIF}}_{apparent}$, ${\mathrm{DIF}}_{rate}$, and $\Delta f_{inertia}$ are apparent DIF, pure rate DIF, and strength enhancement caused by inertia effects, respectively. $\ddot{\varepsilon }$, $\rho_{s}$, $d_{s}$, and $l_{s}$ denote axial strain acceleration, density, the diameter of the specimen, and the initial specimen length, respectively. $k_{1}$, $k_{2}$, and $k_{3}$ are the material parameters.

**Figure 1:**
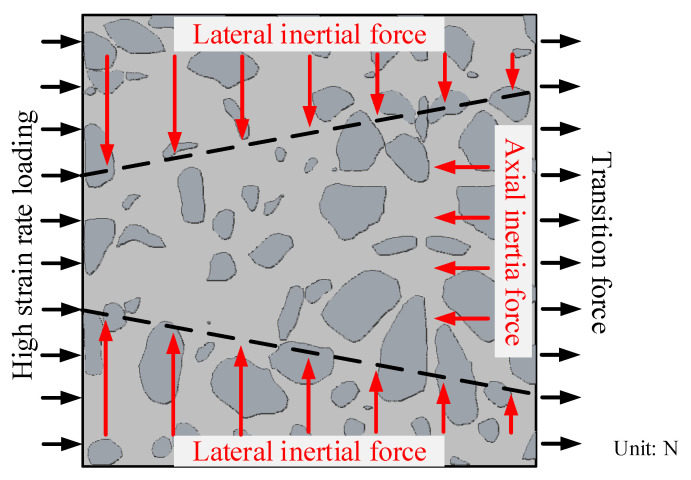
Inertia mechanism of specimen.

Under the high strain rate, the particles in the specimen will obtain axial acceleration, i.e., axial inertial force. In addition, lateral inertial force is also generated due to the influence of Poisson’s ratio. The macroscopic resistance of concrete must balance the actual failure force, axial inertia force, and lateral inertia force [27], as shown in Figure 1 and Equation (11).

(11) $$Q=f(\sigma_{f})+I(ma,\mu_{d})$$

where Q is the macroscopic resistance, $f(\sigma_{f})$ is a function of the actual failure stress, $I(ma,\mu_{d})$ is a function of the inertial force, m is the quality of the particle, a is the acceleration of the particle, and $\mu_{d}$ is the dynamic Poisson’s ratio of the specimen.

Based on the previous research results, we propose a DIF model for high strain rate, as reflected in the following formula:

(12) $$\mathrm{DIF}=K_{1}\log_{10}(\dot{\varepsilon }/\varepsilon_{s})+K_{2}\ddot{\varepsilon }+K_{3}$$

where $K_{1}$, $K_{2}$, and $K_{3}$ are constants. The model considers the strain rate effect and the inertia effect. In order to verify the correctness of the model, SHPB experiments of mortar and concrete were carried out in this paper.

## 3. Experimental Research

### 3.1. Prepare for the Experiment

**Figure 2:**
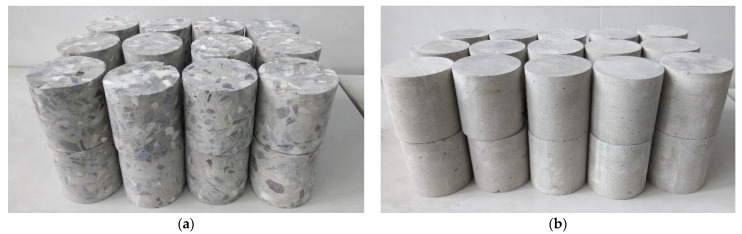
Specimens for SHPB test: (**a**) Mortar, (**b**) Concrete.

Ordinary Portland cement (OPC) and potable laboratory tap water were used for the experiments. Conventional crushed stone with particle sizes between 8 and 12 mm and natural river sand with particle sizes between 0.25 and 0.5 mm were employed as coarse and fine aggregates, respectively. The concrete was mixed at the proportion of 0.52:1:1.67:2.47 (water/cement/sand/aggregate) and subsequently set standing for 24 h. The mold of the specimen was removed, and the specimen was placed in a constant temperature (20 °C) and humidity (95%) curing box for 28 days. The specimens were drilled and polished to smooth the end face after curing. The mortar specimens have the same composition and preparation as the cement paste in the concrete. The diameter and height of the mortar and concrete specimens used for the SHPB test were 71 × 71 mm, respectively, as shown in Figure 2.

**Figure 3:**
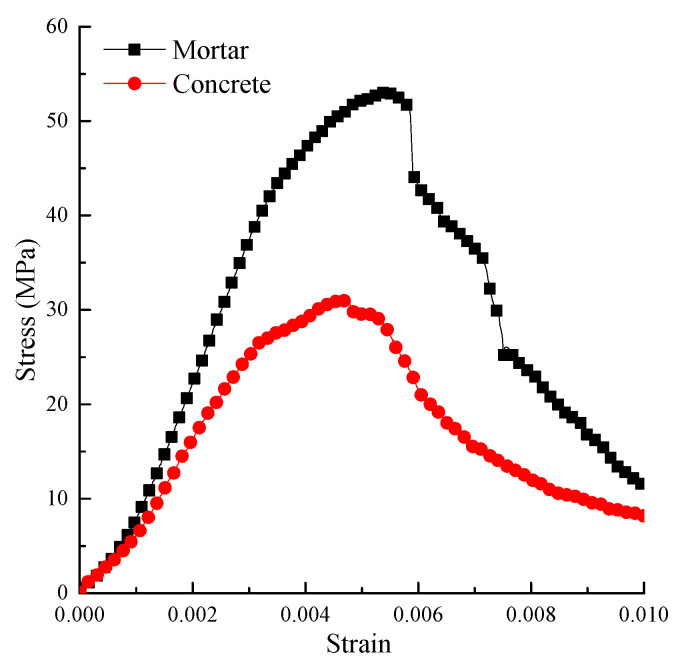
Stress–strain curve of the quasi-static test.

A quasi-static test was conducted before the dynamic load test. An RMT-150B multi-functional full-automatic rigid rock servo testing machine was used for the static load test. The specimen radius and height were 50 and 100 mm, respectively. There were 3 test specimens of mortar and concrete, respectively. The average peak stresses of mortar and concrete specimens were 53.06 and 31.61 MPa, respectively, and their standard deviations were 2.31 and 1.91 MPa, respectively. In order to analyze the dynamic response characteristics of the two kinds of materials, the strain rates of mortar and concrete were extracted in the test. The average strain rates of mortar and concrete specimens were 1.03 × 10^−5^ and 1.12 × 10^−5^/s, respectively, and their standard deviations were 4.71 × 10^−8^ and 2.16 × 10^−7^, respectively. The stress–strain curves of mortar and concrete specimens, whose stress peak value is close to the average value, are shown in Figure 3.

From Figure 3, the compressive strength and elastic modulus of mortar are significantly greater than that of concrete under quasi-static load. This is because the aggregate of the concrete specimen has little effect under low strain rate, while the interfacial transition zone significantly reduces the bearing capacity. The compressive strength of mortar and concrete was 53.07 and 31.23 MPa, respectively. The strain rates of mortar and concrete were 1.03 × 10^−5^ and 1.12 × 10^−5^/s, respectively.

### 3.2. SHPB Experimental Instrument

Split Hopkinson Pressure Bar (SHPB) test technology is the most important and reliable test method to study the mechanical properties of materials under a high strain rate. The basic working principle of the SHPB test device [1] is that when the striker bar is pushed by air pressure to hit the incident pressure bar, an incident wave is produced in the bar. When the incident wave reaches the end, a portion is reflected to form a reflected wave. Another portion will penetrate the specimen and enter the transmission bar to become a transmission wave. The calculation formulas for strain $\varepsilon_{S}(t)$, strain rate ${\dot{\varepsilon }}_{S}(t)$, and stress $\sigma_{S}(t)$ of the specimen in the test are as follows:

(13) $$\varepsilon_{S}(t)=-\frac{C_{0}}{l_{0}}\int_{0}^{t}[\varepsilon_{I}(t)-\varepsilon_{R}(t)-\varepsilon_{T}(t)]dt$$

(14) $$\sigma_{S}(t)=\frac{AE_{0}}{2A_{S}}[\varepsilon_{I}(t)+\varepsilon_{R}(t)+\varepsilon_{T}(t)]$$

(15) $${\dot{\varepsilon }}_{S}(t)=-\frac{C_{0}}{l_{0}}[\varepsilon_{I}(t)-\varepsilon_{R}(t)-\varepsilon_{T}(t)]$$

where: $C_{0}$ is the P-wave velocity of compression bar, $l_{0}$ is the length of the specimen, $A,A_{S}$ are the cross-sectional areas of compression bar and specimen, respectively, $E_{0}$ is the elastic modulus of the compression bar, and $\varepsilon_{I}(t)$, $\varepsilon_{R}(t)$, and $\varepsilon_{T}(t)$ are the strain signals of the incident, reflected waves, and transmission waves, respectively.

**Figure 4:**
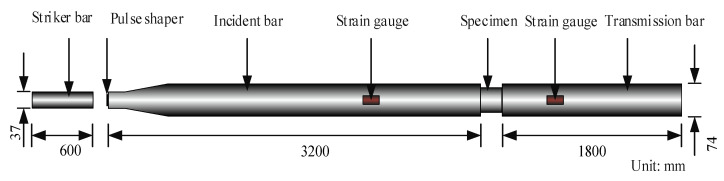
Schematic diagram of SHPB test.

To obtain accurate and reliable data, a tapered incident bar with a diameter of 74 mm was used in the SHPB system as shown in Figure 4. The steel bars had a Young’s modulus $E_{0}$ = 200 GPa and Poisson’s ratio υ = 0.3.

There is severe waveform dispersion in the large diameter SHPB test. To prolong the rise time of the incident wave and filter its high-frequency oscillation, a pulse shaper was fixed on the end face of the incident bar in contact with the bullet [42,43]. The rectangular impact pulse was transformed into a triangular pulse to lengthen its rising edge by the pulse shaper. The wave dispersion can also be reduced by placing the strain gauge on the transmission bar as close to the specimen as possible. Vaseline was applied on both end faces of the sample to reduce the influence of the radial inertia effect by reducing friction. The brass and rubber shapers were tested with no specimen to examine the effect of different pulse shapers. Both shapers had a 20 mm diameter.

**Figure 5:**
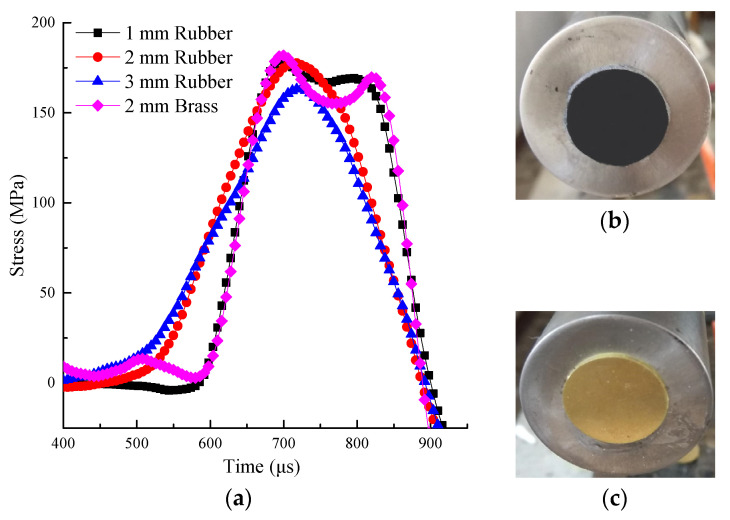
The waveforms of incident waves with different pulse shapers: (**a**) Stress time history curve, (**b**) Rubber sharper, (**c**) Brass sharper.

The SHPB test was carried out with 2 mm thick brass shaper and rubber shaper. The test results show that the shaping effect of rubber shaper is better. In order to obtain a better shaping effect, SHPB tests were carried out on rubber shapers with thicknesses of 1, 2, and 3 mm, respectively. The impact air pressure was 0.3 MPa, and the waveform test with no specimen is shown in Figure 5.

Figure 5 depicts the stress wave curves monitored in the incident bar. The positive and negative values of the curve indicate the direction of stress. It shows that the waveforms obtained by the brass shaper and 1 mm thick rubber shaper are relatively similar and both rectangular. The waveforms obtained by 2 and 3 mm thick rubber shapers are triangular waveforms, and the rise time is also long. When the incident waveform is a half-sine wave, the constant strain rate loading of the specimen is realized, and the inertia effect is greatly reduced [44]. At 0.25 MPa impact pressure, the 1 mm thickness rubber shaper was selected, and the 2mm thickness rubber shaper was selected in other cases.

### 3.3. SHPB Experimental

**Figure 6:**
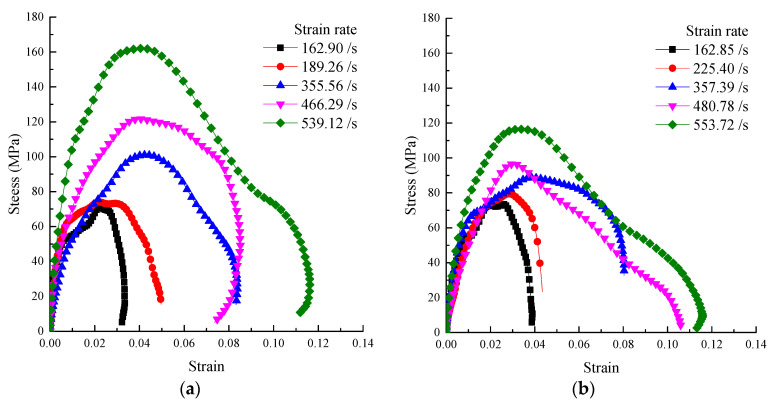
Stress–strain curve: (**a**) Mortar, (**b**) Concrete.

The striker bar can obtain different initial velocities by different impact air pressures. The selected five groups of impact pressures were 0.25, 0.3, 0.4, 0.5, and 0.6 MPa. When the impact air pressure is 0.25 MPa, a 1 mm thickness rubber shaper was selected because the current signal cannot be collected by a 2 mm thickness rubber shaper. A 2 mm thick rubber shaper was used in other cases. Each group was subjected to three impact tests. The data with a large error were removed. Then, a typical stress–strain curve was selected from each group to plot, as shown in Figure 6.

Both graphs have the same scale on the x and y axes for comparison in Figure 6. It shows that the strain rate increases the peak stress of mortar and concrete samples. It indicates that mortar and concrete specimens have a noticeable strain rate effect. The corresponding strain rate time history curve is shown in Figure 6.

**Figure 7:**
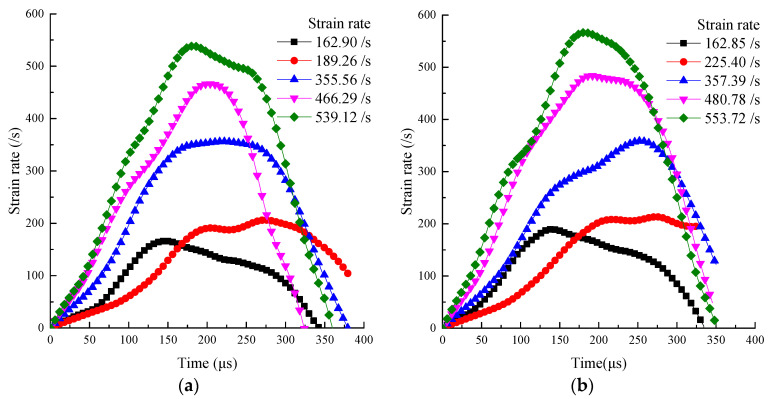
Strain rate time history curve: (**a**) Mortar, (**b**) Concrete.

Figure 7 shows that high strain rates were obtained for the specimens by the SHPB test with a large-diameter bar. The maximum mortar and concrete strain reached 539.12 and 553.72/s, respectively. When the impact air pressure was 0.25 MPa, a 1 mm thickness rubber shaper was selected because a 2 mm thickness rubber shaper cannot collect the current signal. When the impact pressure was 0.25 MPa, the thickness of the rubber shaper was 1 mm, and when the slope of the rising edge of the strain rate time history curve was greater than at 0.30 MPa impact pressure, the thickness was 2 mm. Under a high strain rate, the duration of the constant strain rate is short, and the inertia effect is dominant.

When comparing the impact pressure of 0.25 and 0.30 MPa in Figure 4, Figure 5 and Figure 6, the rubber shaper thickness was 1 mm and 2 mm, respectively. Although the impact pressure of 0.25 MPa is less than that of 0.30 MPa, the slope of the rising edge of the waveform obtained by 1 mm thick shaper is greater than that obtained by 2 mm. In the corresponding strain rate time history curve, the slope of the rising section with a 1 mm thick shaper is also greater than that obtained with a 2 mm. The rising slope of the strain rate time history curve is defined as the strain acceleration [40]. The strain acceleration is directly proportional to the inertial effect [27]. It shows that different strain accelerations can be obtained by the thickness of the pulse shaper. The peak stress of the two cases is close in Figure 5, indicating that the inertia effect increases the dynamic strength of the sample. When the thickness of rubber shaper is the same, the peak stress and strain acceleration increase significantly with the impact pressure. It can also be seen from Figure 6 that under high strain rate, the duration of the constant strain rate is short, and the inertia effect is dominant.

### 3.4. Dynamic Uniaxial Strength Criterion

**Figure 8:**
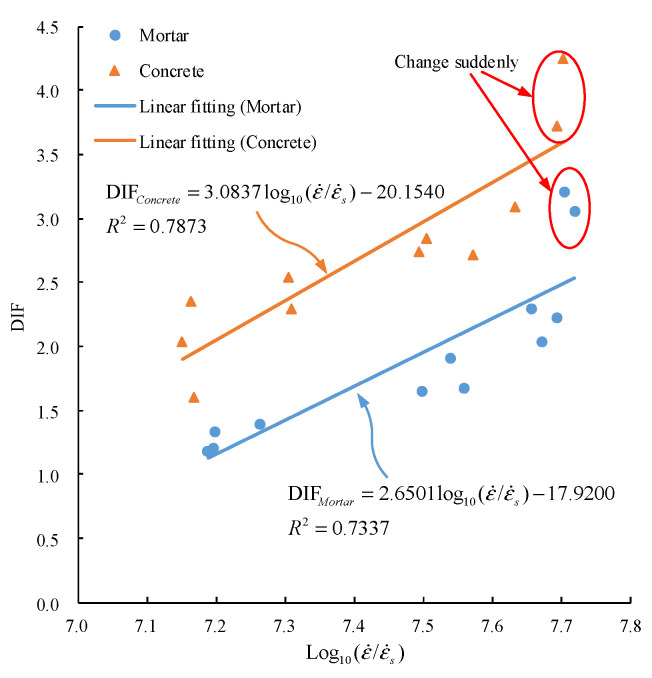
Variation of DIF with logarithm of strain rate.

According to the analysis in Section 2.1, it is common to describe the relationship between DIF and strain rate by logarithmic function under the high strain rate. Therefore, the logarithmic function was adopted to fit the relationship between DIF and strain rate. The fitting results are shown in Figure 8.

In Figure 8, the relationship between DIF and strain rate of mortar and concrete is expressed by a logarithmic function as:

(16) $$\left\{ \begin{array}{l} {\mathrm{DIF}}_{Mortar}=2.6501\log_{10}(\dot{\varepsilon }/{\dot{\varepsilon }}_{s})-17.9200,\;R^{2}=0.7337\\ {\mathrm{DIF}}_{Concrete}=3.0837\log_{10}(\dot{\varepsilon }/{\dot{\varepsilon }}_{s})-20.1540,\;R^{2}=0.7873 \end{array}\right.$$

where ${\mathrm{DIF}}_{Mortar}$, ${\mathrm{DIF}}_{Concrete}$ are dynamic increase factors of mortar and concrete, respectively. The logarithmic function can express the trend relationship between DIF and strain rate. The strain rate effect of concrete is more sensitive than that of mortar by comparing the coefficient of $\log_{10}(\dot{\varepsilon }/{\dot{\varepsilon }}_{s})$. When $\log_{10}(\dot{\varepsilon }/{\dot{\varepsilon }}_{s})>7.7$, the DIF of mortar and concrete has a noticeable sudden change (see the mark in Figure 8). Therefore, the logarithmic strain rate cannot accurately describe DIF under a high strain rate.

**Figure 9:**
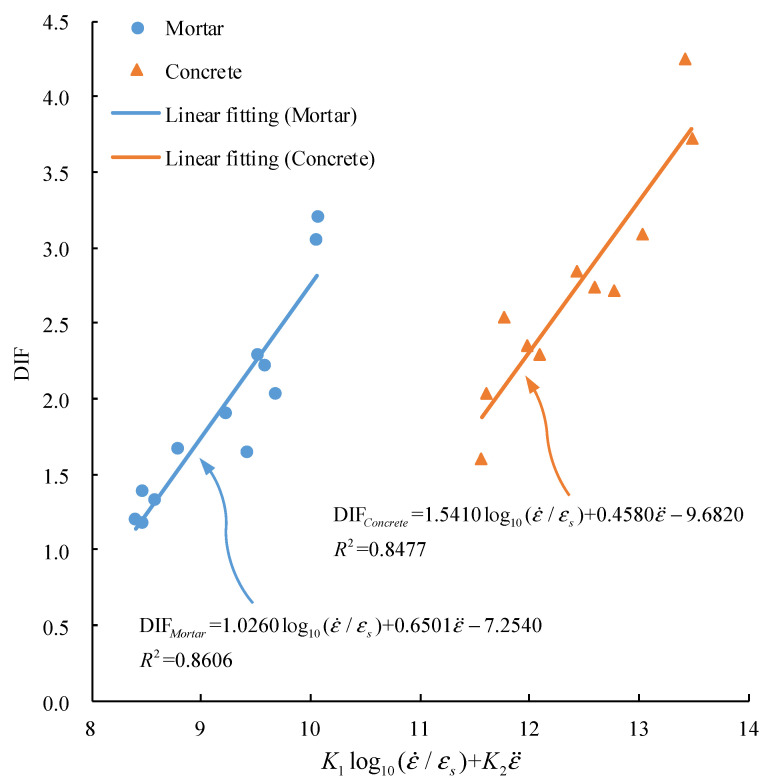
Variation of DIF with the logarithm of strain rate and strain acceleration.

Next, the inertia effect was considered. SHPB test data were fitted by Equation (12). The abscissa was set as $K_{1}\log_{10}(\dot{\varepsilon }/\varepsilon_{s})+K_{2}\ddot{\varepsilon }$ to express the fitting relationship, and the fitting results are shown in Figure 9.

In Figure 9, the DIF fitting function of mortar and concrete considering strain rate and strain acceleration is as follows:

(17) $$\left\{ \begin{array}{l} {\mathrm{DIF}}_{Mortar}=1.0260\log_{10}(\dot{\varepsilon }/\varepsilon_{s})+0.6501\ddot{\varepsilon }-7.2540,\;R^{2}=0.8606\\ {\mathrm{DIF}}_{Concrete}=1.5410\log_{10}(\dot{\varepsilon }/\varepsilon_{s})+0.4580\ddot{\varepsilon }-9.6820,\;R^{2}=0.8477 \end{array}\right.$$

When compared with Equation (16), the fitting degree is improved. It indicates that Equation (12) is feasible to fit the DIF of mortar and concrete under a high strain rate. When comparing the $\log_{10}(\dot{\varepsilon }/\varepsilon_{s})$ coefficient in Equation (17), the strain rate effect of concrete is more sensitive than that of mortar, which is consistent with the above analysis. When compared with the $\ddot{\varepsilon }$ coefficient, the inertia effect of mortar is more sensitive than that of concrete. Therefore, the strain rate effect of the material is more sensitive, but the inertia effect is not necessarily more sensitive.

## 4. Research on Energy Utilization and Fragmentation Morphology

### 4.1. Energy Utilization Research

The energy utilization was studied to compare the effect of strain rate on the energy utilization of mortar and concrete in large-diameter SHPB tests. The energy calculation formula of stress waves is:

(18) $$\left\{ \begin{array}{l} W_{I}(t)=E_{0}C_{0}A_{S}\int_{0}^{t}\varepsilon_{I}^{2}(t)dt\\ W_{T}(t)=E_{0}C_{0}A_{S}\int_{0}^{t}\varepsilon_{T}^{2}(t)dt \end{array}\right.$$

where $W_{I}$ is incident energy and $W_{T}$ is the transmission energy.

The calculation formula of energy utilization η is:

(19) $$\eta =\frac{W_{T}}{W_{I}}\cdot 100\%$$

**Figure 10:**
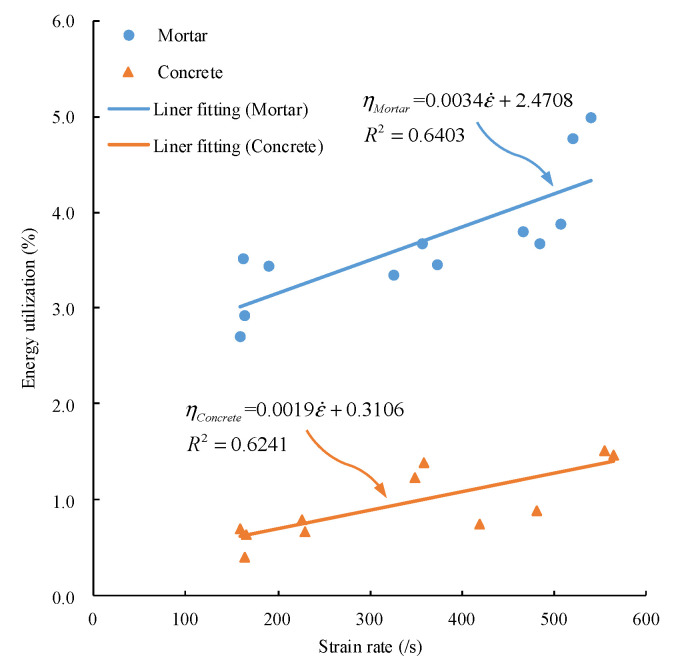
Relation between energy utilization and strain rate.

Figure 10 represents the energy utilization of mortar and concrete. The energy utilization of mortar and concrete increases with the strain rate, but the increase of mortar is faster. Under the same strain rate, the energy utilization of mortar is higher than that of concrete. Under the impact compression of large diameter SHPB, the energy utilization of mortar and concrete specimens is relatively low, and the highest is only 4.99%.

### 4.2. Fragmentation Morphology Research

The fracture morphology is an important aspect of evaluating the impact potentiality of concrete materials [45,46]. The pore sizes of classifying screens selected in this test were 2.0, 5.0, 10.0, 20, and 40 mm. The broken specimens were sieved into six groups with particle size ranges of 0.0–2.0, 2.0–5.0, 5.0–10.0, 10.0–20.0, 20.0–40.0, and 40–71 mm (71 mm is the diameter of the sample before fragmentation), respectively. The weighing instrument was a high-precision electronic scale with a measuring range of 1 kg and an accuracy of 0.1 g.

**Figure 11:**
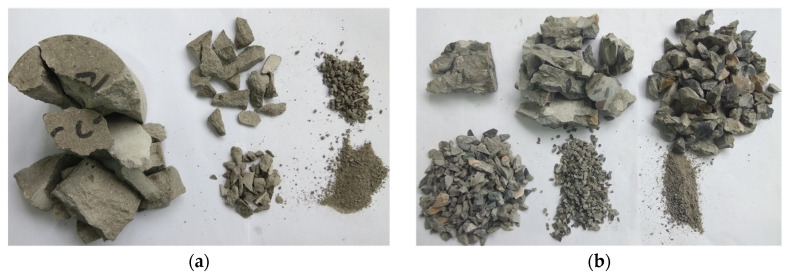
Fragmentation morphologies of mortar and concrete: (**a**) Mortar (466.29/s), (**b**) Concrete (480.78/s).

After impact, the broken specimens were collected, classified, and screened individually. First, the classifying screens were stacked from high to low according to the pore size. Then, the broken specimens were placed on the sieve with the largest mesh size on the uppermost layer, so that the specimens with different fragmentation degrees could be separated according to size. After screening, the fragmentation on each sieve was placed on the electronic scale for weighing, and the measurement results were recorded one by one. The screened fragmentations of mortar and concrete are shown in Figure 11.

Figure 11 shows the final fragmentation morphologies of mortar and concrete under the similar strain rate. It can be seen that the fragmentation morphologies of mortar and concrete specimens under impact load are different. The mortar sample was cracked along the axial direction. Although the mortar specimen was penetrated by cracks, the strip fragment still had high strength in the loading direction. The strips after impact splitting can still bear the impact load on the bar as a whole. However, the fragmentation degree of concrete specimen was very large, and the cracks mostly occurred in the interface transition zone (ITZ) between mortar and aggregate. From the fact that the strength of concrete was lower than that of mortar, the aggregate plays a small role in dynamic loading, so the ITZ reduces the strength of concrete.

To quantify the fragmentation degree of the specimen, the average fragment size of the broken specimen was adopted. The calculation formula is as follows:

(20) $$\bar{X}=\frac{\sum n_{i}X_{i}}{\sum n_{i}}$$

where $\bar{X}$ is the average fragment size of the broken specimen in mm, $X_{i}$ is the average size of specimen fragmentation retained on the classifying screen of class *i*, in mm, and $n_{i}$ is the proportion of fragment mass with an average size $X_{i}$ in %.

**Figure 12:**
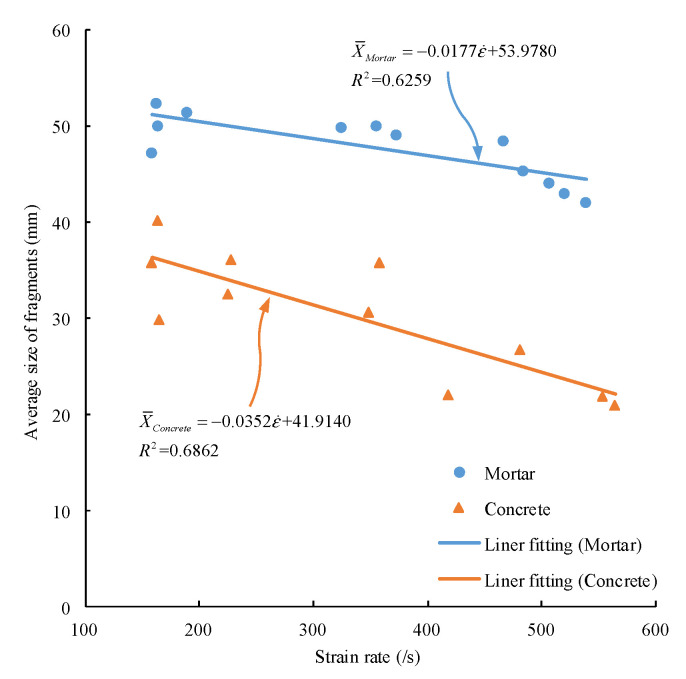
Relation between average block size and strain rate of mortar and concrete.

The median values of the average size of the fragmentation on each classifying screen were taken according to the sieve diameter, which are 1, 3.5, 7.5, 15, 30, and 55.5 mm. The relationship between the average fragment size of mortar and concrete and strain rate is shown in Figure 12.

In Figure 12, ${\bar{X}}_{Mortar}$ and ${\bar{X}}_{Concrete}$ represent the average fragment size of mortar and concrete, respectively. With the increase of strain rate, the average fragment size of mortar and concrete decreases, but that of the concrete decreases faster. Under the same strain rate, the average fragment size of mortar is larger than that of concrete. The average fragment size of mortar is 42.03–52.36 mm, and that of concrete is 20.89–40.21 mm. Therefore, mortar is better than concrete in the storage performance of elastic strain energy, indicating that the impact failure ability of mortar is stronger than that of concrete.

## 5. Discussion

Under high strain load, axial acceleration will be obtained by the grains in the specimen. The lateral acceleration will be obtained due to the Poisson effect. In the study of inertial effect, the lateral inertial force is often considered, while the axial inertial force is often ignored. Although the axial acceleration is consistent with the bearing capacity direction of the specimen, it is caused by the uneven stress of the sample. It is closely related to the slope of the rising edge of the loading waveform, and independent from the constant strain rate of the specimen. The axial acceleration is not a part of the real strength of the specimen. Therefore, the axial inertial force should also be taken into account in the study of inertial effect. The lateral strain or dynamic Poisson’s ratio should be taken as the monitored object during the SHPB test.

The inertial effect was studied by theoretical analysis or numerical simulation, but the data extraction of strain acceleration was often ignored. In the SHPB test, it was found that the rising edge of the incident wave can be changed by rubber shapers with different thicknesses, and the inertia effect can be changed accordingly. Therefore, the control variable method can be used in the indoor test, that is, the strain rate and strain acceleration can be controlled respectively to study the inertial effect. The test can be repeated in the laboratory. One of the highlights of this paper is in establishing a DIF model considering inertial effect by considering strain rate and strain acceleration. In the future research, digital image correlation (DIC), CT scanning, and other technologies could be used to retrieve the strain, crack, and damage of the specimen under inertial effect [47,48]. It is helpful to deeply understand the inertial effect, establish an accurate dynamic model, and provide a theoretical basis for rock dynamics theory, disaster warnings, and safety assessments.

## 6. Conclusions

In this paper, the large-diameter SHPB tests on concrete and mortar were performed, the inertia effect was studied. The conclusions are as follows:

(1) The macroscopic resistance of concrete material is composed of the actual failure force, axial inertia force, and lateral inertia force. The dynamic growth factor (DIF) model was established. The DIF model comprehensively considers the influence of strain rate on the actual dynamic strength of concrete materials and the influence of strain acceleration on inertial effect.
(2) With the increase of bullet impact velocity, the influence of inertia effect becomes greater and greater. The strain rate effect of concrete is more sensitive than that of mortar, but the inertia effect of mortar is more sensitive than that of concrete.
(3) With the increase of strain rate, the energy utilization of mortar and concrete increases, while the average fragment size decreases. Under the same strain rate, the energy utilization rate, average fragment size, and impact potentiality of mortar are higher than that of concrete.

## Data Availability

Not applicable.
